# Overexpression of Osteopontin-a and Osteopontin-c Splice Variants Are Worse Prognostic Features in Colorectal Cancer

**DOI:** 10.3390/diagnostics14192108

**Published:** 2024-09-24

**Authors:** Daniella Mattos, Murilo Rocha, Josiane Tessmann, Luciana Ferreira, Etel Gimba

**Affiliations:** 1 Hemato-Oncology Molecular Program, National Institute of Cancer, 23rd Red Cross Square, 6th Floor, Rio de Janeiro 20230-130, RJ, Brazil; dani95mattos@gmail.com; 2 Biomedical Science Graduation Program, Fluminense Federal University, Rua Professor Hernani Pires de Melo, 101, Niterói 24210-130, RJ, Brazil; 3 Cellular and Molecular Oncobiology Program, National Institute of Cancer, Rio de Janeiro 20231-050, RJ, Brazil; rocham.2@gmail.com (M.R.); josiwt@hotmail.com (J.T.); 4 Departamento de Genética, Instituto de Ciências Biológicas e da Saúde, Universidade Federal Rural do Rio de Janeiro, BR-465, Km 07, Seropédica, Rio de Janeiro 23897-000, RJ, Brazil; 5 Departamento de Ciências da Natureza, Humanities and Healthy Institute, Fluminense Federal University, Recife Street, Bela Vista, Rio das Ostras 28895-532, RJ, Brazil

**Keywords:** osteopontin, colorectal cancer, splice variants

## Abstract

**Background**: Osteopontin (OPN) is a glycoprotein involved in various physiological and pathological processes, and its aberrant expression in cancer cells is closely linked to tumor progression. In colorectal cancer (CRC), OPN is overexpressed, but the roles of its splice variants (OPN-SVs), OPNa, OPNb, and OPNc, are not well understood. This study aimed to characterize the expression patterns of OPN-SVs and their potential diagnostic and prognostic implications in CRC using transcriptomic data deposited in TSVdb and TCGA. **Methods**: The expression patterns of each OPN-SV were analyzed using transcriptomic data deposited in TSVdb and TCGA, which were correlated to patient data available at cBioPortal. **Results**: Bioinformatic analysis revealed that OPNa, OPNb, and OPNc are overexpressed in CRC samples compared to non-tumor samples. Notably, OPNa and OPNc are overexpressed in CRC stages (II, III, and IV) compared to stage I. Higher levels of OPNa and OPNc transcripts are associated with worse overall survival (OS) and shorter progression-free survival (PFS) in CRC patients. Additionally, the expression of OPNa, OPNb, and OPNc is correlated with BRAFV600E mutations in CRC samples. **Conclusions**: These findings suggest that OPNa and OPNc, in particular, have potential as diagnostic and prognostic biomarkers, paving the way for their further evaluation in CRC diagnosis and prognosis.

## 1. Introduction

Colorectal cancer (CRC) is one of the most common tumors worldwide, according to the Global Cancer Observatory [1,2,3], despite advances in CRC treatment and early detection [4]. Searching for new molecules and therapeutic targets that may improve CRC screening and the patient’s prognosis is key in this tumor type [5].

Osteopontin (OPN) is among the several gene products aberrantly expressed in CRC [6], and increased OPN plasma levels are potentially related to the occurrence of metastasis and poor prognosis in CRC patients [6,7]. Increased OPN transcriptional levels have been observed in colon tumors in relation to non-tumor tissues [8]. Moreover, OPN has been proposed as a putative biomarker and molecular target for new therapeutic strategies against this tumor [5,9,10]. Recent data based on single-cell RNA sequencing (scRNA-seq) analysis have shown that CRC samples presenting macrophages expressing OPN are negatively correlated with lymphocyte infiltration and predicted poor patient survival, besides contributing to resistance to PD- L1 blockade immunotherapy [11,12], and also conferring CRC cells proliferative and invasive properties [13]. Other authors found that in liver metastatic CRC samples, OPN was markedly enriched in the cancer-associated fibroblasts (CAFs) and macrophages, being significantly associated with worse clinical outcomes in CRC patients [14]. Also, a high OPN expression was found in a subset of macrophages with strong senescence-associated secretory phenotype (SASP) features. Using CRC tissues, it was discovered that OPN-positive macrophages were surrounded by a large number of senescent tumor cells in high-grade tumors [13].

Most studies related to OPN in tumor progression have evaluated total OPN (tOPN), which corresponds to the sum of all OPN variants, including those generated by post-transcriptional and post-translational mechanisms [15]. Notably, the OPN primary transcript undergoes alternative splicing, generating at least five splice variants (OPN-SVs), named as OPNa, OPNb, OPNc, OPN4, and OPN5 [15,16,17,18,19]. The functional roles of OPNa, OPNb, and OPNc in human tumors have been explored in several studies, with findings that are specific to cell type and tissue. However, the roles of the recently identified OPN4 and OPN5 remain unclear [19,20]. A single report characterized OPN5 expression in the mouse primary and metastatic breast tumors [21]. Although the roles and expression profile of tOPN in CRC progression have been previously described [6,8,22], studies regarding the OPN-SVs expression patterns and their prognostic impacts in CRC are scarce. More recent data regarding the OPNc variant in CRC point that it can modulate the response to 5-fluorouracil (5-FU) by promoting the survival of colon cancer cells [23]. Once abnormal splicing and cancer-associated splice variants play critical roles in cancer progression [24], a better understanding of each specific OPN-SV is crucial to comprehend how it may modulate CRC biology.

Osteopontin expression has also been previously associated with BRAF mutations, particularly in thyroid tumors [25,26]. BRAF mutations are also known as genetic alterations in CRC, occurring in 5–10% of cases, and are associated with poor prognosis [27,28]. Among CRC patients harboring BRAF mutations, the BRAFV600E is the most common, accounting for ~80% [29]. BRAF encodes a serine/threonine protein kinase of the RAF family and is involved in the regulation of the RAS-RAF-MEK-ERK signaling [30], which is activated in tumor growth and progression, including proliferation, angiogenesis, invasion, and metastasis [31,32].

In this scenario, by using the TSVdb and TCGA transcriptional databases, this study aimed to characterize the expression patterns of the OPN-SVs OPNa, OPNb, and OPNc in CRC samples, and their putative diagnostic and prognostic implications, besides their association with clinicopathological and molecular features.

## 2. Materials and Methods

### 2.1. Data Acquisition and Features

We used OPN-SVs transcript data of CRC samples from The Cancer Genome Atlas projects (TCGA) [33]. Using the TCGA Splicing Variants Database ‘TSVdb’ web tool [34], we downloaded OPN-SVs transcript data of CRC from the TSVdb data portal (http://tsvdb.com/) on 12 October 2022. The corresponding clinicopathological data from the TCGA dataset including progression-free survival (PFS), sex, age, KRAS mutation, BRAF mutation, vascular invasion, and radiation therapy data were downloaded from the website of the cBioPortal for Cancer Genomics (http://www.cbioportal.org/) at 12 April 2023.

We analyzed the OPNa, OPNb, and OPNc transcriptional levels in non-tumor colon and rectal (*n* = 51) and in CRC (*n* = 382) samples, including 287 tissue samples from colon adenocarcinoma (COAD) and 95 samples from rectal adenocarcinoma (READ). Among these samples, the survival data for 25 of these samples was not available; thus, 357 samples were analyzed regarding the survival analysis. Survival analysis was performed using the Kaplan–Meier method and log-rank test. The cutoff values of OPN-SV expression levels were determined using the Cutoff Finder tool (https://molpathoheidelberg.shinyapps.io/CutoffFinder_v1, accessed on 20 March 2023), classifying each sample as low or high for each OPN-SV group.

### 2.2. Statistical Analysis

The statistical tests were performed using the SPSS software package (IBM SPSS Statistics version 28). One-way ANOVA or Kruskal–Wallis were used for mean and median comparisons, respectively. The Kaplan–Meier method and log-rank test were used to assess associations between variables, overall survival, and PFS. Univariate and multivariate analyses using the Cox-proportional hazards model were performed to evaluate potential prognostic factors for OS. A statistically significant difference was defined when *p*-value < 0.05.

## 3. Results

### 3.1. OPN-SVs Are Overexpressed in CRC Tissues

**Figure 1:**
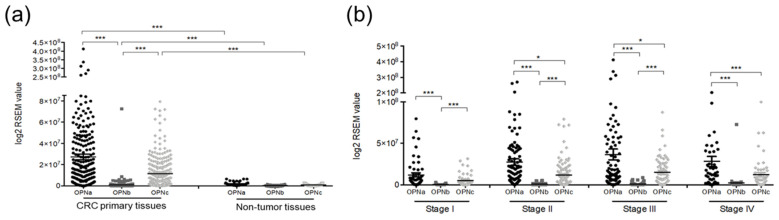
Expression levels of OPN-SV in colon carcinoma tissues: (**a**) OPN-SV expression levels in CRC tissues versus colorectal non-tumor tissues. The transcriptional levels of OPNa (black dots), OPNb (gray squares), and OPNc (gray diamonds) were assessed using TCGA data. Scatter plots the median transcriptional levels in CRC samples (*n* = 379), comprising 285 COAD tissue samples and 94 READ tissue samples, as well as non-tumor samples (*n* = 51) from TCGA. (**b**) mRNA levels of OPNa, OPNb, and OPNc were analyzed in CRC samples shown in panel (**a**), representing CRC stages (I–IV). Expression levels in (**a**,**b**) are derived from RNAseq data downloaded from TCGA, showing gene-level transcription estimates, as log2 transformed RSEM normalized counts. Statistical analysis was conducted using the Kruskal–Wallis test (**a**,**b**), with significance values adjusted by the Bonferroni correction for multiple comparisons. * *p* ≤ 0.05, *** *p* ≤ 0.001 indicate statistically significant values.

We analyzed the transcriptional patterns of OPNa, OPNb, and OPNc in both non-tumor and CRC samples. These OPN-SVs are expressed at significantly higher levels in CRC samples compared to non-tumor tissues (*p* < 0.05), with OPNb being the least expressed of the three variants (Figure 1a). We also analyzed the transcriptional levels of OPNa, OPNb, and OPNc across different stages of CRC (I–IV) (Figure 1b). In the majority of tumor stages, OPNa was found to be expressed at higher levels compared to OPNc and OPNb (*p* < 0.05). Regarding the expression of the isoforms individually, OPNa showed a significant difference across stages I and II (*p* = 0.004), I and III (*p* = 0.003), and I and IV (*p* = 0.017). The same was observed for the OPNb (I and II (*p* = 0.012), I and III (*p* = 0.009), I and IV (*p* = 0.014)), and for the OPNc (I and II (*p* = 0.004), I and III (*p* = 0.004), and I and IV (*p* = 0.016)).

### 3.2. OPN-SVs Overexpression in CRC Tissues Is Associated with Poor Survival Rates

**Figure 2:**
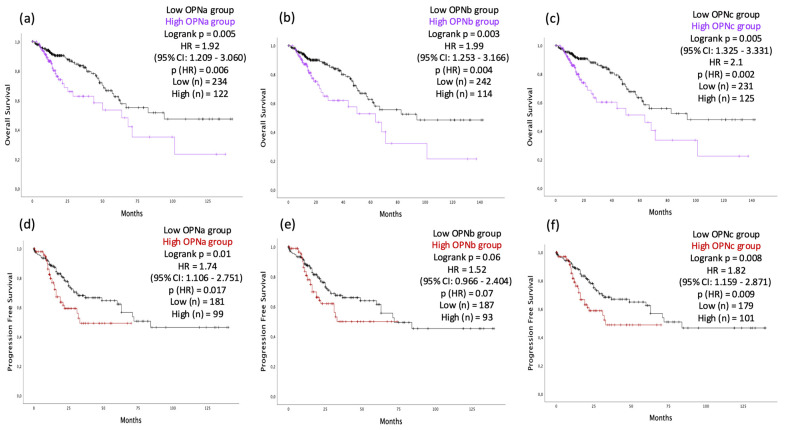
Comparison of overall survival (OS) and progression-free survival (PFS) rates between the low and high OPN-SV expression levels in CRC patient samples. Overall survival is represented by Kaplan–Meier plots where the color lines represent the low (black line) and high (purple line) OPNa (**a**), OPNb (**b**), and OPNc (**c**) expression levels in CRC patient samples. The log-rank test was used to analyze differences in survival curves between the groups. PFS is represented by Kaplan–Meier plots where the color lines represent the low (black line) and high (red line). PFS time is represented by Kaplan–Meier curves, where the color lines represent the low (black line) and high (red line) OPNa (**d**), OPNb (**e**), and OPNc (**f**) expression levels in CRC patient samples. Log-rank *p* < 0.05 was considered statistically significant. HR: hazards ratio; CI = confidence interval.

To further explore how the expression levels of each OPNa, OPNb, or OPNc could impact CRC prognosis, we evaluated the association of their expression levels with CRC patient overall survival rates (OS). CRC samples (*n* = 357), including tissues from COAD and READ, were categorized into low- and high expression levels for each OPN-SV. CRC samples with elevated OPN-SV transcript levels exhibited poorer overall survival (OS) rates. Specifically, higher levels of OPNa were associated with an HR of 1.92 (95% CI: 1.209 to 3.060, *p* = 0.006, Figure 2a); OPNb with an HR of 1.99 (95% CI: 1.253 to 3.166, *p* = 0.004, Figure 2b); and OPNc with an HR of 2.1 (95% CI: 1.325 to 3.331, *p* = 0.002, Figure 2c). Furthermore, a prognostic model for progression-free survival (PFS) revealed that patients with overexpression of OPNa and OPNc had a significantly shorter PFS. Specifically, OPNa overexpression was associated with an HR of 1.74 (95% CI: 1.106 to 2.751, *p* = 0.01, Figure 2d); OPNb with an HR of 1.52 (95% CI: 0.966 to 2.404, *p* = 0.07, Figure 2e); and OPNc with an HR of 1.82 (95% CI: 1.159 to 2.871, *p* = 0.009, Figure 2f). The median PFS was 33, 35, and 33 months for patients with high levels of OPNa, OPNb, and OPNc, respectively. In contrast, patients with low levels of these isoforms had a median PFS of 84, 72, and 84 months for OPNa, OPNb, and OPNc, respectively.

### 3.3. High Levels of OPNa Are Associated with PFS

To evaluate if the clinicopathological and/or molecular features from this CRC cohort are independent predictors for PFS, we performed a univariate logistic regression model (Table 1). There were significant differences in PFS between patients displaying low and high OPNa transcript levels (*p* = 0.044). Patients whose OPNa transcript levels were higher exhibited a shorter PFS (HR and 95%CI: 1.63, 1.01–2.64, *p* = 0.044) (Table 1).

### 3.4. OPN-SV Overexpression Is Associated with BRAFV600E Mutation

To further explore the relevance of OPN-SV expression levels in CRC phenotypes, we compared OPN-SV mRNA levels among CRCs with various clinicopathological and molecular characteristics, including sex, age, *KRAS* mutation, *BRAF* mutation, vascular invasion, and radiation therapy. We found that CRC patients with the *BRAFV600E* mutation exhibited significantly higher transcript levels of OPNa, OPNb, and OPNc compared to those with wild-type patients presenting *BRAF* (*p* = 0.007, *p* = 0.03 and *p* = 0.04, respectively) (Table 2).

## 4. Discussion

Understanding the expression patterns of OPN-SVs and their specific impact on CRC diagnosis and prognosis remains limited. This study aimed to compare the expression patterns of OPNa, OPNb, and OPNc between non-tumor and colon and rectal cancer samples. Additionally, we sought to investigate the prognostic potential of these OPN-SVs by examining the association between their expression levels and various molecular and clinicopathological features.

We found that OPNa, OPNb, and OPNc are overexpressed in CRC primary tumors compared to non-tumor samples, with OPNa being particularly elevated in primary tumor tissue across all CRC stages. Notably, OPNa expression levels were highest in the advanced stages of CRC (II and III). Despite the fact that more than two-thirds of CRC patients undergo radical surgery, 30% to 50% of those with stage II or stage III tumors inevitably experienced tumor relapse, manifesting as locoregional recurrence and distant metastasis [35]. These findings suggest that OPN-SVs, particularly OPNa, may have diagnostic potential and could play roles in risk stratification and CRC progression. To further validate these data, we conducted early functional assays trying to better understand how these OPN splice variants might be related to worse prognostic features in CCR, thereby validating the bioinformatics analysis. We characterized the expression patterns of OPNa, OPNb, and OPNc in colon carcinoma cell lines (Caco-2, HT-29, and HCT-116), which showed higher OPNa transcript levels compared to the other two OPN-SVs. OPNa showed the highest levels in the poorly differentiated colon carcinoma cell lines (HCT-119 and HT-29), followed by the well-differentiated Caco-2. Based on the expression patterns of OPN-SVs in colon carcinoma cell lines and CRC tumor samples, we investigated how each OPN-SV could modulate CRC cell growth rates. Caco-2 cells ectopically overexpressing OPNa evidenced higher growth rates than those overexpressing OPNb, OPNc, and EV control in a time-course analysis (Supplementary Materials). Similar to what has been observed for OPNa in other cancers, such as thyroid carcinoma [36], and in lung cancer [37], these data support the notion that OPNa could be valuable in CRC diagnosis and management. It is known that less than half of CRC cases are diagnosed at an early stage; however, early detection significantly increases the chances of cure [2,38,39]. Therefore, developing new methods for early CRC detection could significantly reduce mortality associated with this tumor type. Among the many biomarkers being explored for CRC diagnosis, those that can be detected non-invasively represent the ideal options. Previous studies have demonstrated that OPN-SV transcripts can be identified in cancer serum samples [40], suggesting their potential as a non-invasive strategy for early CRC detection. Furthermore, targeting OPN-SV in therapeutic approaches might be more effective compared to tOPN, as discussed by other researchers [5].

To investigate the association between OPN-SV expression levels and CRC prognostic features, we analyzed overall survival (OS) and progression-free survival (PFS) rates for each OPN-SV. Patients with higher mRNA levels of OPNa, OPNb, and OPNc had poorer prognoses compared to those with lower expression levels, with elevated OPNa, OPNb, and OPNc significantly associated with reduced OS. Additionally, CRC tissue samples with high expression levels of OPN-SVs, particularly OPNa and OPNc, were strong predictors of shorter PFS. In a meta-analysis, Liu and collaborators [41] found that high tOPN expression levels in patients with non-small cell lung cancer were significantly associated with poor overall survival (OS) and unfavorable progression-free survival (PFS) [41]. Similarly, tOPN has been proposed as a promising prognostic biomarker for PFS in renal cell carcinoma, in conjunction with other cytokines (e.g., IL-8) [42]. Another study demonstrated that the tOPN transcriptional levels are strong indicators of both OS and PFS for patients with clear cell renal carcinoma, based on univariate and multivariate analyses [43]. Additionally, OPN-SV mRNA levels were significantly correlated with adverse clinical outcomes in soft tissue sarcoma [44].

In gastric tumors, high expression levels of OPNb and OPNc have been associated with aggressive clinicopathological features [45]. Similarly, in a prostate cancer model, our group observed that overexpression of OPNc correlates with poor prognostic outcomes [46]. Although our data provide preliminary evidence that these three OPN-SVs could serve as prognostic biomarkers in CRC, further evaluation of their expression levels in additional cohorts is needed to validate their prognostic utility. The three OPN-SVs differ in their gene structures: OPNa is the full-length isoform, including all seven exons; OPNb lacks exon 5; and OPNc lacks exon 4. Exon 4 encodes a proline-rich region, a transglutaminase site, and several phosphorylation sites, while exon 5 encodes additional protein phosphorylation sites. Both exons 4 and 5 are crucial for OPN post-translational modifications (PTMs) and its interaction with OPN receptors. PTMs such as Ser/Thr phosphorylation, tyrosine sulfation, and glycosylation contribute to the molecular weight variability of OPN, which ranges from 44 to 75 kDa. These modifications lead to structural and functional changes that enhance our understanding of OPN’s diverse roles. The structural differences among these variants may influence their distinct roles in tumor progression, as extensively reported [16,47]. A common mutation in CRC is found in the *BRAF* gene, particularly the V600E substitution, which occurs in approximately 10% of patients with metastatic CRC. This mutation is associated with poor prognosis in both early and advanced stages of the disease and serves as a predictive marker for treatment response [47,48]. Strategies that combine inhibition of the mitogen-activated protein kinase (MAPK) have shown promising results in treating patients with *BRAFV600E*-mutated metastatic CRC [49]. The BRAF V600E mutation leads to abnormal activation of the MAPK signaling pathway, which is crucial for mediating cellular responses to growth signals. This aberrant activation results in increased expression of oncogenes and decreased expression of tumor suppressors. Numerous studies have emphasized the connection between hyperactivation of this pathway and altered expression of integrin genes of this signaling cascade and altered expression of integrin genes [49]. Based on these observations, we then hypothesize that BRAF mutations may drive the expression of these OPN-SV, as integrin receptors mediate the roles of OPN in cancer cells. Osteopontin expression has been linked to *BRAF* mutations in thyroid tumors [25,26]. Specifically, our previous research found that OPNa overexpression was significantly associated with *BRAFV600E* mutation in papillary thyroid carcinoma samples [36]. Additionally, overexpression of total OPN, regardless of each specific isoform, has been shown to promote CRC cell migration, invasion, and anchorage-independent growth in the presence of *KRAS* mutation [50]. In our current study, we also observed a strong association between high OPN-SV transcript levels and the *BRAFV600E* mutation. We hypothesize that in CRC, these splicing isoforms and mutant-*BRAF* may work together to activate signaling pathways that drive rectal and colon carcinogenesis and tumor progression. Future research should explore how the BRAFV600E mutation impacts OPN-SV expression levels and associated signaling pathways, as well as functionally evaluate the effects on tumor progression. Further work should also investigate how the overexpression of these OPN-SV modulates various aspects of CRC progression and biology.

## 5. Conclusions

We conclude that OPNa expression levels in CRC samples show diagnostic potential. Additionally, the OPN-SVs, particularly OPNa and OPNc, are associated with worse prognostic features in CRC. These findings suggest that OPN-SVs could serve as valuable biomarkers, offering new opportunities for enhancing CRC diagnosis and prognosis.

## Figures and Tables

**Table 1:** Predictive factors for progression-free survival among CRC patient’s cohort.

Variable		Univariate Analysis		
		HR	95% CI	*p* Value
OPNa	Low	1.000		
	High	1.636	(1.013–2.644)	0.044
OPNb	Low	1.000		
	High	1.168	(0.723–1.886)	0.525
OPNc	Low	1.000		
	High	1.179	(0.732–1.898)	0.499
Sex *	Male	1.000		
	Female	0.688	(0.408–1.162)	0.162
Age *	<45	1.000		
	≥45	1.273	(0.772–2.101)	0.345
*KRAS* mutation *	Absent	1.000		
	Present	1.528	(0.509–4.586)	0.450
*BRAF* mutation *	Absent	1.000		
	Present	2.044	(7.047–5.595)	0.164
Vascular invasion *	Absent	1.000		
	Present	1.697	(0.967–2.977)	0.065
Radiation Therapy *	Absent	1.000		
	Present	1.237	(0.306–4.993)	0.765

* Clinicopathological data was missing in some cases included in the series.

**Table 2:** Association between OPNa, OPNb, and OPNc expression and the clinicopathological characteristics in CRC.

Variable		OPNa Expression		*p* Value	OPNb Expression		*p* Value	OPNc Expression		*p* Value
		Low n (%)	High n (%)		Low n (%)	High n (%)		Low n (%)	High n (%)	
Sex *	**Male**	83 (64.8%)	45 (35.2%)	0.801	86 (67.2%)	42 (32.8%)	0.835	80 (62.5%)	48 (37.5%)	0.788
	**Female**	97 (63.4%)	56 (36.6%)		101 (66%)	52 (34%)		98 (64.1%)	55 (35.9%)	
Age *	**<45**	90 (68.2%)	42 (31.8%)	0.49	94 (71.2%)	38 (28.8%)	0.364	89 (67.4%)	43 (32.6%)	0.487
	**≥45**	161 (64.7%)	88 (35.3%)		166 (66.7%)	83 (33.3%)		159 (63.9%)	90 (36.1%)	
*KRAS* mutation *	**Absent**	21 (72.4%)	8 (27.6%)	0.707	21 (72.4%)	8 (27.6%)	0.934	20 (69%)	9 (31%)	0.708
	**Present**	19 (67.9%)	9 (32.1%)		20 (71.4%)	8 (28.6%)		18 (64.3%)	10 (35.7%)	
*BRAF* mutation *	**Absent**	34 (77.3%)	10 (22.7%)	**0.007**	35 (79.5%)	9 (20.5%)	**0.035**	32 (72.7%)	12 (27.3%)	**0.046**
	**Present**	14 (46.7%)	16 (53.3%)		17 (56.7%)	13 (43.3%)		15 (50%)	15 (50%)	
Lymphovascular invasion *	**Absent**	144 (63.4%)	83 (36.6%)	0.772	155 (68.3%)	72 (31.7%)	0.248	143 (63%)	84 (37%)	0.702
	**Present**	63 (61.8%)	39 (38.2%)		63 (61.8%)	39 (38.2%)		62 (60.8%)	40 (39.2%)	
Vascular invasion *	**Absent**	162 (65.1%)	87 (34.9%)	0.520	171 (68.7%)	78 (31.3%)	0.301	161 (64.7%)	88 (35.3%)	0.433
	**Present**	47 (61%)	30 (39%)		48 (62.3%)	29 (37.7%)		46 (59.7%)	31 (40.3%)	
Radiation Therapy *	**Absent**	112 (63.3%)	65 (36.7%)	0.213	117 (66.1%)	60 (33.9%)	0.282	113 (63.8%)	64 (36.2%)	0.550
	**Present**	9 (81.8%)	2 (18.2%)		9 (81.8%)	2 (18.2%)		8 (72.7%)	3 (27.3%)	

* Clinicopathological data were missing in some cases included in the series.

## Data Availability

The data presented in this study are available on request from the corresponding authors.

## Supplementary Materials

The following supporting information can be downloaded at: https://www.mdpi.com/article/10.3390/diagnostics14192108/s1, Figure S1: OPN-SV expression levels in colon carcinoma cell lines; Figure S2: Ectopic OPN-SV expression in Caco-2 cells and cell growth rates.
