# Supplementary material for: Overexpression of Osteopontin-a and Osteopontin-c Splice Variants Are Worse Prognostic Features in Colorectal Cancer
Source: Diagnostics (Basel). 2024 Sep 24;14(19):2108. doi: 10.3390/diagnostics14192108 (PMC11475046; doi:10.3390/diagnostics14192108)
Supplement: Supplementary file 1 [file diagnostics-14-02108-s001.zip › diagnostics-3171094-supplementary.pdf]

Supplementary Material

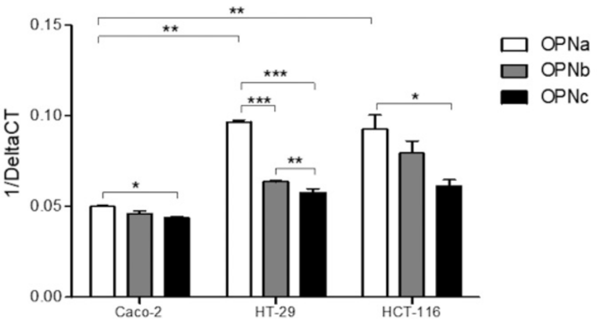

Figure S1: OPN-SV expression levels in colon carcinoma cell lines. The mRNA levels of OPNa, OPNb and OPNc in colon carcinoma cell lines (HCT-116, HT-29 and Caco-2) were analyzed by RT-qPCR assays. The bar graphs represent the transcriptional levels of OPN-SV in each cell line by using the 1/ΔCT method. Statistical analysis was performed using ANOVA test (a). * $p \leq 0.05$, ** $p \leq 0.01$, *** $p \leq 0.001$ values are statistically significant.

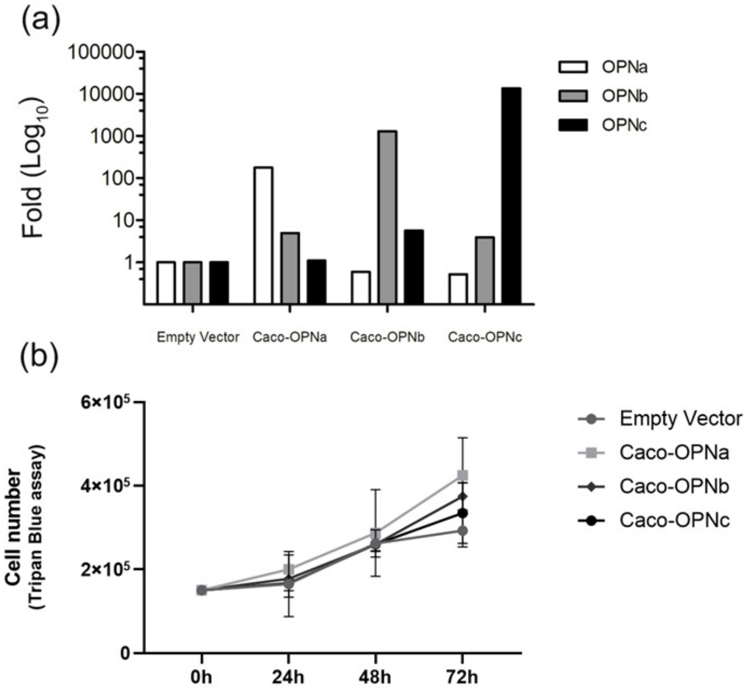

Figure S2: Ectopic OPN-SV expression in Caco-2 cells and cell growth rates. Growth rates in response to OPNa, OPNb or OPNc overexpression in Caco-2 cell line. (a) Expression levels of each OPN-SV in Caco-2 cell line tested by RT-qPCR assays. The bar graphs represent the transcriptional levels of OPNa, OPNb and OPNc splice variants by using the 2-ΔΔCT method (b) Growth analysis of OPN-SV overexpression in Caco-2 cell lines, according to trypan blue cell counting at the indicated time points (0 h, 24 h, 48 h and 72 h). This experiment was repeated three times and statistical analysis was performed using the One-way ANOVA test. P values were $p \geq 0{,}05$.
